# Association of sodium intake and major cardiovascular outcomes: a dose-response meta-analysis of prospective cohort studies

**DOI:** 10.1186/s12872-018-0927-9

**Published:** 2018-10-19

**Authors:** Yaobin Zhu, Jing Zhang, Zhiqiang Li, Yang Liu, Xing Fan, Yaping Zhang, Yanbo Zhang

**Affiliations:** 1 Cardiovascular Surgery II, Beijing Children’s Hospital, Capital Medical University, National Center for Children’s Health, Beijing, 100045 China; 2 Pediatric Heart Center, Beijing Anzhen Hospital, Capital Medical University, Beijing, 100029 China; 3 The Heart Center, Beijing Friendship Hospital, Capital Medical University, Beijing, 100050 China; 4 National Clinical Research Center of Cardiovascular Diseases, State Key Laboratory of Cardiovascular Disease, Fuwai Hospital, Chinese Academy of Medical Sciences and Peking Union Medical College, Beijing, 100037 China

**Keywords:** Sodium intake, Cardiovascular outcomes, Dose-response, Meta-analysis, Prospective cohort studies

## Abstract

**Background:**

The association of sodium intake with the risk of cardiovascular morbidity and mortality is inconsistent. Thus, the present meta-analysis was conducted to summarize the strength of association between sodium intake and cardiovascular morbidity and mortality.

**Methods:**

PubMed, Embase, and the Cochrane Library were searched systematically to identify the relevant studies up to October 2017. The effect estimates for 100 mmol/day increase in sodium intake were calculated using 95% confidence intervals (CIs) of cardiac death, total mortality, stroke, or stroke mortality for low (< 3 g/d), moderate (3–5 g/d), or heavy (> 5 g/d) sodium intake, and minimal sodium intake comparison.

**Results:**

A total of 16 prospective cohort studies reported data on 205,575 individuals. The results suggested that an increase in sodium intake by 100 mmol/d demonstrated little or no effect on the risk of cardiac death (*P* = 0.718) and total mortality (*P* = 0.720). However, the risk of stroke incidence (*P* = 0.029) and stroke mortality (*P* = 0.007) was increased significantly by 100 mmol/day increment of sodium intake. Furthermore, low sodium intake was associated with an increased risk of cardiac death (*P* = 0.003), while moderate (*P* < 0.001) or heavy (*P* = 0.001) sodium intake was associated with an increased risk of stroke mortality.

**Conclusions:**

These findings suggested that sodium intake by 100 mmol/d increment was associated with an increased risk of stroke incidence and stroke mortality. Furthermore, low sodium intake was related to an increased cardiac death risk, while moderate or heavy sodium intake was related to an increased risk of stroke mortality.

## Background

Cardiovascular diseases (CVD) are the major causes of mortality and morbidity in the general population, accounting for approximately 17.5 million deaths worldwide. The World Health Organization (WHO) estimated over 30% of all the deaths worldwide annually due to CVDs [1]. Several studies have recommended several lifestyle factors such as intake of yogurt [2], dietary magnesium [3], nuts [4], whole grains [5], dietary fibers [6], milk [7], and saturated and trans unsaturated fatty acids [8] that prevent the progression of CVD. However, the relatively high residual risk for CVD should be addressed, and it is necessary to understand the association of individual dietary components with CVD at the population level to alter the dietary habits and improve the health conditions.

Dietary sodium intake has been documented as a modifiable risk factor for blood pressure, which in turn, is associated with the progression of CVD [9–12]. Currently, WHO recommends a sodium intake of < 2 g/d, which is largely based on the small and short-term clinical trials that evaluated the effect of modest salt reduction on blood pressure in general population [13]. However, the effect of dietary sodium intake on subsequent cardiovascular morbidity and mortality is limited and inconclusive.

Several prospective studies have indicated that long-term interventions aiming at sodium reduction may reduce the risk of CVD [14, 15]. Moreover, the results of another prospective study did not show any correlation between sodium intake and CVD [16]. Furthermore, several studies suggested that high sodium intake may decrease the risk of cardiac death [17, 18]. Hence, clarifying the optimal daily intake of sodium is essential in the general population as it has not yet been determined. Herein, we attempted to investigate the available prospective cohort studies on a large-scale to determine the association of sodium intake and cardiovascular morbidity and mortality.

## Methods

### Data sources, search strategy, and selection criteria

This study was conducted and reported according to the Preferred Reporting Items for Systematic Reviews and Meta-Analysis (PRISMA) guidelines [19]. Studies with a prospective cohort design evaluating the impact of sodium intake and the risk of major cardiovascular outcomes, without any language bias (English or another language), were included in this meta-analysis. Electronic databases, such as PubMed, Embase, and Cochrane Library were searched for literature published up to October 2017. The core search terms used were “dietary salt” OR “sodium” AND (“cardiovascular disease” OR “stroke” OR “cardiac death” OR “mortality” OR “death” OR “CVD” OR “myocardial infarction” OR “coronary events”) AND “clinical trials” AND “human”. The reference lists from potentially relevant studies were searched to select the additional eligible studies. Parameters such as the study topic, design, participants’ status, exposure, and reported outcomes were employed to identify the relevant studies.

The literature search and study selection was conducted by two authors independently, and any inconsistencies were settled by group discussion until a consensus was reached. The inclusion criteria for the studies were as follows: (1) prospective cohort design; (2) evaluation of the impact of sodium intake and the risk of major cardiovascular outcomes; (3) reported at least 1 of the following outcomes: cardiac death, total mortality, stroke, or stroke mortality; (4) the data should provide the effect estimates, such as relative risk (RR), hazard ratio (HR), or odds ratio (OR,) and 95% confidence intervals (CIs) or crude data that compared the different categories of sodium intake vs. the minimal sodium intake with respect to 100 mmol/day increments and the risk of major cardiovascular outcomes. All the retrospective observational studies were excluded as various confounding factors could bias the results.

### Data collection and quality assessment

The data collected from the eligible studies included the first author’s name, publication year, country, sample size, age at baseline, percentage male, assessment of exposure, reference category of sodium intake, reported outcomes, follow-up duration, and covariates in the fully adjusted model. Also, the crude data on the number of cases/persons or person-years, effect of different exposure categories, and the 95% CIs were collected. In addition, the effect estimates that were maximally adjusted for potential confounders, if the study provided several adjusted effect estimates, were selected.

The comprehensive Newcastle–Ottawa Scale (NOS) has been partially validated for evaluating the quality of the observational studies in the meta-analysis, and hence, was used to evaluate the quality of the study method [20]. The NOS evaluated the quality of the observational studies based on selection (4 items), comparability (1 item), and outcome (3 items). The maximum score was 9, and the minimum score was 0 (Additional file : Table S1). The data were extracted and quality assessed by 2 authors independently, and any inconsistencies were referred to the original studies by an additional author.

### Statistical analysis

To evaluate the impact of sodium intake and the risk of major cardiovascular outcomes, we collected the effect estimates (RR, HR, or OR) and the 95% CIs or the relevant crude data from each study. The summary RRs and 95% CIs for the low (< 3 g/d), moderate (3–5 g/d), or heavy (> 5 g/d) sodium intake vs. and the minimized intake of sodium and the risk of major cardiovascular outcomes were calculated using the random-effects model [21, 22]. Next, we evaluated the estimates of the RR associated with every 100 mmol/day increase in sodium by the generalized least-squares method for trend estimation [23], assuming the presence of a linear relationship between the natural logarithm of the RR and increasing sodium intake. The mid-point for closed categories and median for open categories putatively determined each sodium intake category, presuming a normal distribution for sodium intake. The summary RRs for 100 mmol/day increase in sodium intake was calculated using random-effects meta-analysis [22, 24].

The heterogeneity among the included studies was assessed using the I^2^ and Q statistic, and a *P*-value < 0.10 was considered as significant heterogeneity [25, 26]. Subgroup analyses were conducted for cardiac death, total mortality, and stroke according to publication year, sample size, percentage male, assessment of exposure, following-up duration, and with or without adjusted body mass index (BMI), smoking, alcohol, previous CVD, diabetes mellitus (DM), physical activity (PA), and level of potassium. The *P*-value between subgroups was evaluated by chi-square test and meta-regression [27]. A sensitivity analysis was evaluated the impact of individual studies by removing individual study from the meta-analysis [28]. Funnel plot and Egger [29] and Begg [30] tests investigated the outcomes that were also used to evaluate any potential publication bias. All the reported *P*-values are 2-sided, and *P* < 0.05 was considered statistically significant. The statistical analyses were conducted using STATA software (version 10.0; Stata Corporation, College Station, TX, USA).

## Results

### Literature search

**Fig. 1:**
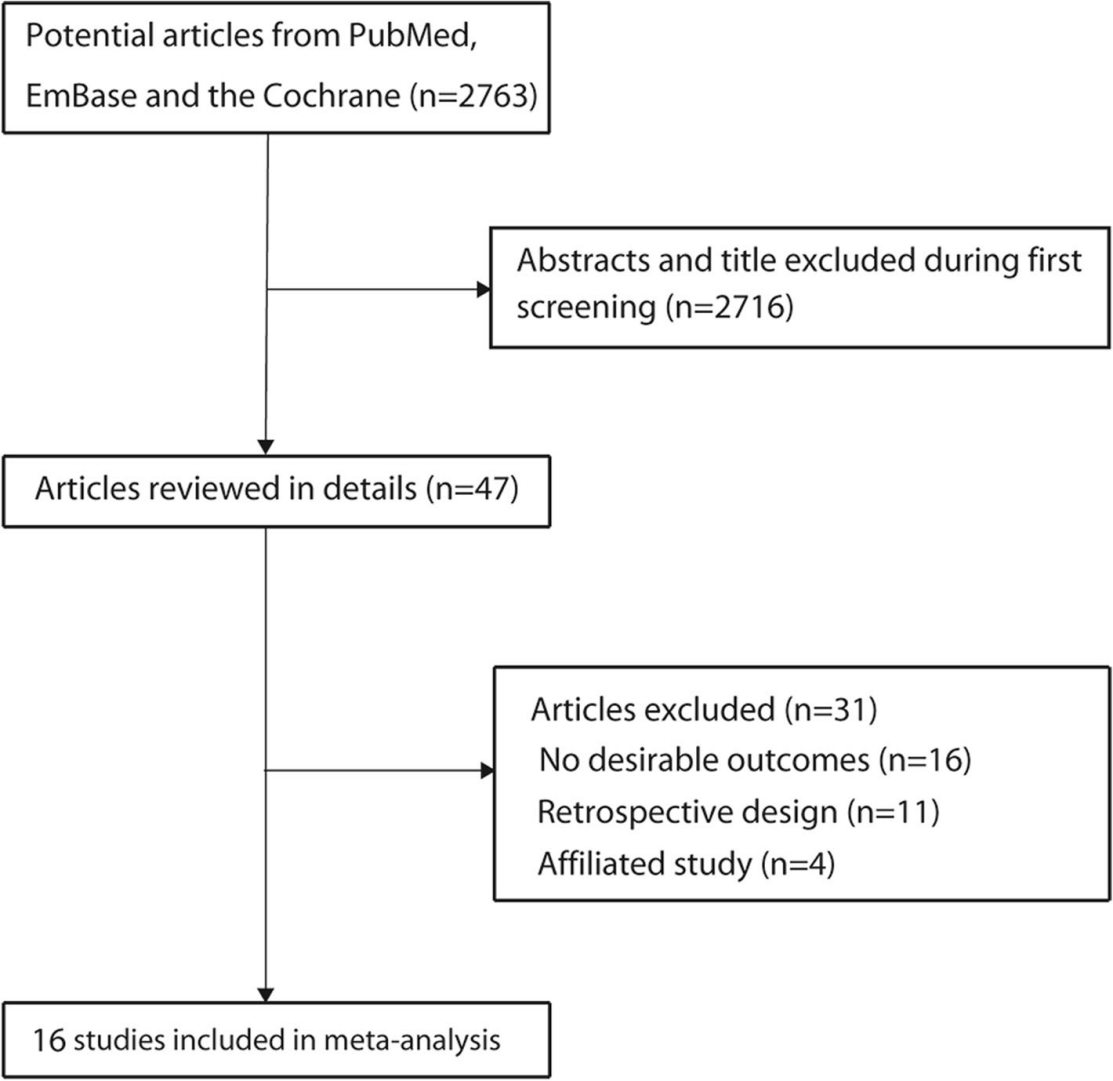
Schematic representation of the study selection process

The results of the study selection process were presented in Fig. 1. A total of 2763 articles were identified in the initial electronic search. Of these, 2716 were excluded as they were duplicates and irrelevant studies. Thus, a total of 47 potentially eligible studies were selected. After a detailed evaluation, 16 prospective cohort studies were selected for the final meta-analysis [14, 16, 18, 31–43]. A manual search of the reference lists of these studies did not yield any new eligible studies. The general characteristics of the included studies were presented in Table 1.

**Table 1:** Baseline characteristic of studies included in this meta-analysis

Study	Publication year	Country	Sample size	Age at baseline	Percentage male (%)	Assessment of exposure	Reference category of sodium intake	Reported outcomes	Follow-up (year)	Adjusted factors	NOS score
Alderman [31]	1995	US	2937	53.0	64.7	24 h urine collection	Quartile I	Stroke	3.5	Age, and race	7
Tunstall-Pedoe [32]	1997	Scotland	11,629	40.0–59.0	49.5	24 h urine collection	129.6 mmol/day	Cardiac death, total mortality	7.6	Age	8
He [33]	1999	US	9485	25.0–74.0	38.9	24 h urine collection	Quartile I	Cardiac death, stroke, stroke mortality, total mortality	19.0	Age, sex, race, SBP, SC, BMI, DM, diuretic use, PA, education, alcohol, smoking, EI	9
Tuomilehto [34]	2001	Finland	2436	25.0–64.0	48.2	24 h urine collection	< 159 mmol/day	Cardiac death, stroke, total mortality	8.0	Age, study year, smoking, HDL, SBP, and BMI	9
Nagata [35]	2004	Japan	29,079	> 35.0	49.6	FFQ		Stroke mortality	7.0	Age, EI, marital status, education, BMI, smoking, alcohol, PA, hypertension, DM, and intake of protein, potassium, and vitamin E	9
Cohen [36]	2008	US	8699	> 30.0	44.9	FFQ		Cardiac death, total mortality	8.7	Age, sex, race, education, added table salt, PA, alcohol, smoking, DM, history of cancer, SBP, TC, dietary potassium, weight, antihypertensive drug	9
Larsson [37]	2008	Finland	26,556	50.0–69.0	100	FFQ		Stroke	13.6	Age, supplementation group, smoking, BMI, SBP and DBP, SC, serum HDL, DM and CVD, PA, alcohol and EI	8
Umesawa [14]	2008	Japan	58,730	40.0–79.0	39.4	FFQ		Cardiac death, stroke mortality	12.7	BMI, smoking, alcohol, history of hypertension, DM, menopause, HRT, PA, educational, perceived mental stress, calcium, and potassium intake	9
Ekinci [38]	2011	Australia	638	64.0	56.0	24 h urine collection		Cardiac death, total mortality	9.9	Age, sex, previous CVD, eGFR, atrial fibrillation, SBP, DM duration	6
Stolarz-Skrzypek [18]	2011	Belgium	3595	40.9	47.3	24 h urine collection		Cardiac death, stroke, total mortality	7.9	Study population, sex, age, BMI, SBP, potassium excretion, antihypertensive drug, smoking, alcohol, DM, TC, and educational	9
Yang [16]	2011	US	12,267	> 20.0	48.1	FFQ		Cardiac death, total mortality	14.8	Sex, race/ethnicity, educational, BMI, smoking, alcohol, TC, HDL, PA, family history of CVD, and EI	9
O’Donnell [39]	2011	Canada	28,880	> 55.0	70.6	24 h urine collection		Cardiac death, stroke, total mortality	4.8	Age, sex, race/ethnicity, history of stroke or MI, creatinine, BMI, comorbid vascular risk factors, treatment allocation, fruit and vegetable, PA, SBP, and urinary potassium	7
Gardener [40]	2012	US	2657	69.0	36.0	FFQ		Stroke	10.0	Age, sex, race/ethnicity, education, alcohol, smoking, PA, EI, total fat, saturated fat, carbohydrates, protein, DM, hypercholesterolemia, hypertension, previous CVD, BMI	9
Mills [41]	2016	US	3757	57.8	55.6	FFQ		Stroke	6.8	Age, sex, race, clinic site, education; waist circumference, BMI, smoking; alcohol, PA, LDL, glucose; history of CVD; use of antidiabetic, lipid-lowering, and BP- lowering medications, urinary creatinine excretion, baseline estimated GFR	9
Kalogeropoulos [42]	2015	US	2642	74.6	48.8	FFQ		Total mortality	10.0	Age, sex, race, BMI, smoking, PA, previous CVD, pulmonary disease, DM, depression, BP, heart rate, electrocardiogram abnormalities, and serum glucose, albumin, creatinine, and SC	9
Horikawa [43]	2014	Japan	1588	58.7	52.5	FFQ		Total mortality	7.0	Age, sex, BMI, HbA1c, DM duration, LDL, HDL, log-transformed triglycerides, insulin, lipid-lowering agents, smoking, alcohol, EI, and PA	7

*BMI body mass index, *BP* blood pressure, *CHD* coronary heart disease, *CVD* cardiovascular disease, *DBP* diastolic blood pressure, *DM* diabetes mellitus, *EI* energy intake, *FFQ* food frequency questionnaire, *eGFR* estimated glomerular filtration rate, *GFR* glomerular filtration rate, *HbA1c* glycated hemoglobin, *HDL* high density lipoprotein, *HRT* hormone replacement therapy, *LDL* low density lipoprotein, *MACEs* major cardiovascular events, *MI* myocardial infarction, *PA* physical activity, *SBP* systolic blood pressure, *SC* serum cholesterol, *TC* total cholesterol

### Study characteristics

A total of 16 prospective cohort studies with 205,575 individuals were eligible for this study. The follow-up period of the participants ranged from 3.5–19.0 years and 638–58,730 individuals were included in each study. A total of 7 studies were conducted in the USA [16, 31, 33, 36, 40–42], 4 in Europe [18, 32, 34, 37], 3 in Japan [14, 35, 43], 1 in Australia [38], and 1 in Canada [39]. Seven studies used 24-h urine collection [18, 31–34, 38, 39], and the remaining 9 studies used food frequency questionnaires (FFQ) to assess the dietary sodium exposure [14, 16, 35–37, 40–43]. The study quality was assessed using the NOS (Table 1). A score of ≥7 was considered as high quality for the study. Overall, 10 studies had a score of 9 [14, 16, 18, 33–36, 40–42], 2 had a score of 8 [32, 37], 3 had a score of 7 [32, 39, 43], and the remaining 1 study had a score of 6 [38].

### Cardiac death

**Fig. 2:**
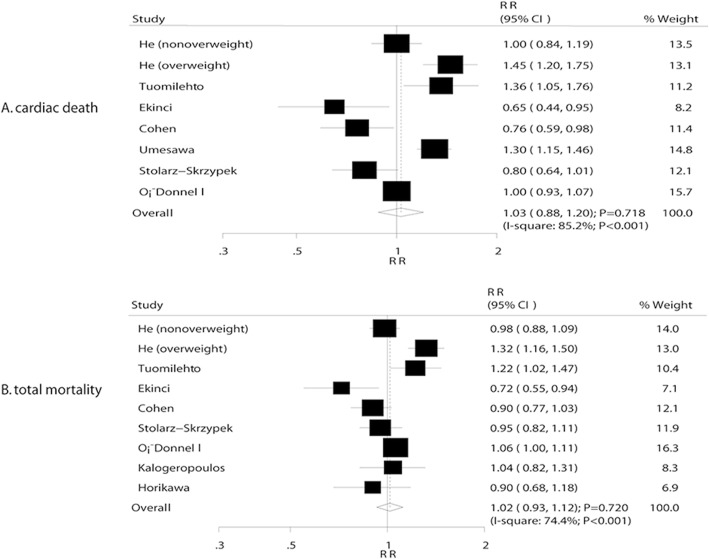
**a** Association between sodium intake and cardiac death. **b** Association between sodium intake and total mortality

A total of 7 studies reported an association between sodium intake and cardiac death. The summary RR showed that a 100 mmol increment per day in sodium intake was not associated with cardiac death (RR, 1.03; 95% CI, 0.88–1.20; *P* = 0.718; Fig. 2a); however, accumulating evidence suggested significant heterogeneity (I^2^ = 85.2%, *P* < 0.001). Sensitivity analysis indicated that the conclusion was unaffected after sequential exclusion of each study from the pooled analysis. Furthermore, the low sodium intake was found to be associated with an increased risk of cardiac death (RR: 1.19; 95% CI: 1.06–1.33; *P* = 0.003), while moderate (RR: 0.91; 95% CI: 0.71–1.15; *P* = 0.421) and heavy (RR: 1.02; 95%CI: 0.92–1.13; *P* = 0.762) sodium intake did not demonstrate a significant effect (Table 2). Subgroup analysis indicated that an increment of 100 mmol/day in sodium intake exerted detrimental effects on cardiac death if the duration of follow-up was ≥10 years (RR: 1.24; 95% CI: 1.02–1.50; *P* = 0.034; Table 3). Conversely, increased sodium intake was associated with the reduced risk of cardiac death if the study was not adjusted for BMI (RR: 0.72; 95% CI: 0.59–0.90; *P* = 0.003; Table 3).

**Table 2:** Summary results for different categories of sodium and subsequent major cardiovascular outcomes

Outcomes	Low sodium	*P* value	Moderate sodium	*P* value	Heavy sodium	*P* value
Cardiac death	1.19 (1.06–1.33)	0.003	0.91 (0.71–1.15)	0.421	1.02 (0.92–1.13)	0.762
Total mortality	1.02 (0.89–1.18)	0.779	0.98 (0.85–1.14)	0.806	1.09 (0.94–1.27)	0.257
Stroke	1.25 (0.85–1.85)	0.260	1.11 (1.00–1.24)	0.058	1.02 (0.93–1.11)	0.720
Stroke mortality	1.20 (0.96–1.50)	0.117	1.50 (1.20–1.88)	< 0.001	1.81 (1.29–2.55)	0.001

**Table 3:** Subgroup analyses for cardiac death

Factor	Subgroup	RR and 95% CI	*P* value	Heterogeneity (%)	*P* value for heterogeneity	*P* value between subgroups
Publication year	Before 2010	1.15 (0.94–1.41)	0.170	82.7	< 0.001	< 0.001
	2010 or after	0.85 (0.67–1.08)	0.174	73.8	0.022	
Sample size	≥ 10,000	1.13 (0.88–1.47)	0.334	92.8	< 0.001	0.513
	< 10,000	0.98 (0.76–1.25)	0.846	84.8	< 0.001	
Percentage male (%)	≥ 60.0	1.00 (0.93–1.07)	1.000	–	–	0.023
	< 60.0	1.03 (0.83–1.26)	0.812	85.7	< 0.001	
Assessment of exposure	FFQ	1.01 (0.59–1.70)	0.982	92.9	< 0.001	0.025
	24 h urine collection	1.03 (0.86–1.23)	0.746	82.2	< 0.001	
Follow-up duration (years)	≥ 10.0	1.24 (1.02–1.50)	0.034	78.0	0.011	< 0.001
	< 10.0	0.91 (0.74–1.11)	0.329	77.4	0.001	
Adjusted BMI	Yes	1.13 (0.96–1.32)	0.146	85.2	< 0.001	< 0.001
	No	0.72 (0.59–0.90)	0.003	0.0	0.506	
Adjusted smoking	Yes	1.09 (0.88–1.33)	0.433	85.3	< 0.001	0.003
	No	0.84 (0.56–1.27)	0.415	78.5	0.031	
Adjusted alcohol	Yes	1.04 (0.83–1.31)	0.735	87.5	< 0.001	0.028
	No	0.99 (0.74–1.34)	0.959	80.4	0.006	
Adjusted Previous CVD	Yes	0.84 (0.56–1.27)	0.415	78.5	0.031	0.003
	No	1.09 (0.88–1.33)	0.433	85.3	< 0.001	
Adjusted DM	Yes	0.98 (0.78–1.23)	0.855	87.4	< 0.001	0.125
	No	1.14 (0.84–1.53)	0.400	80.3	0.024	
Adjusted PA	Yes	1.09 (0.91–1.30)	0.355	87.2	< 0.001	0.113
	No	0.90 (0.59–1.38)	0.636	85.0	0.001	
Adjusted potassium	Yes	0.97 (0.78–1.19)	0.744	88.2	< 0.001	0.070
	No	1.10 (0.82–1.46)	0.533	83.6	< 0.001	

*BMI body mass index, *CI* confidence interval, *CVD* cardiovascular disease, *DM* diabetes mellitus, *FFQ* food frequency questionnaire, *PA* physical activity, *RR* relative risk

### Total mortality

A total of 8 studies reported a correlation between sodium intake and total mortality. However, the results did not reveal any significant association of 100 mmol increments per day in sodium intake with the total mortality risk (RR: 1.02; 95% CI: 0.93–1.12; *P* = 0.720; Fig. 2b). Although substantial heterogeneity was observed in the magnitude of the effect across the studies (I^2^ = 74.4%, *P* < 0.001), after sequential exclusion of each study from pooled analyses, the conclusion was not affected by the exclusion of any specific study. Furthermore, the low (RR: 1.02; 95% CI: 0.89–1.18; *P* = 0.779), moderate (RR: 0.98; 95% CI: 0.85–1.14; *P* = 0.806), and heavy (RR: 1.09; 95% CI: 0.94–1.27; *P* = 0.257) sodium intake was not associated with the risk of total mortality (Table 2). Subgroup analysis indicated that an increment of 100 mmol/day in the sodium intake was associated with an increased risk of total mortality if the sample size was ≥10,000 (RR: 1.06; 95% CI: 1.01–1.12; *P* = 0.029; Table 4), and the percentage male was ≥60.0%.

**Table 4:** Subgroup analyses for total mortality

Factor	Subgroup	RR and 95% CI	*P* value	Heterogeneity (%)	*P* value for heterogeneity	*P* value between subgroups
Publication year	Before 2010	1.09 (0.91–1.30)	0.351	85.2	< 0.001	0.328
	2010 or after	0.96 (0.85–1.08)	0.493	60.2	0.040	
Sample size	≥ 10,000	1.06 (1.01–1.12)	0.029	–	–	0.428
	< 10,000	1.00 (0.89–1.14)	0.958	77.1	< 0.001	
Percentage male (%)	≥ 60.0	1.06 (1.01–1.12)	0.029	–	–	0.428
	< 60.0	1.00 (0.89–1.14)	0.958	77.1	< 0.001	
Assessment of exposure	FFQ	0.93 (0.83–1.04)	0.212	0.0	0.570	0.032
	24 h urine collection	1.05 (0.93–1.17)	0.441	80.4	< 0.001	
Follow-up duration (years)	≥ 10.0	1.11 (0.90–1.37)	0.343	83.9	0.002	0.139
	< 10.0	0.97 (0.87–1.09)	0.616	69.6	0.005	
Adjusted BMI	Yes	1.07 (0.98–1.17)	0.146	68.2	0.004	0.001
	No	0.83 (0.67–1.02)	0.082	51.4	0.151	
Adjusted smoking	Yes	1.04 (0.92–1.17)	0.522	74.5	0.001	0.991
	No	0.89 (0.61–1.30)	0.560	87.0	0.005	
Adjusted alcohol	Yes	1.01 (0.87–1.17)	0.908	80.4	< 0.001	0.471
	No	1.02 (0.87–1.19)	0.804	70.8	0.016	
Adjusted Previous CVD	Yes	0.95 (0.77–1.17)	0.649	74.1	0.021	0.999
	No	1.04 (0.91–1.19)	0.573	78.8	< 0.001	
Adjusted DM	Yes	0.98 (0.85–1.12)	0.716	77.7	< 0.001	0.132
	No	1.11 (0.97–1.25)	0.119	52.4	0.147	
Adjusted PA	Yes	1.04 (0.94–1.15)	0.475	74.2	0.002	0.291
	No	0.96 (0.74–1.24)	0.735	81.4	0.005	
Adjusted potassium	Yes	0.99 (0.88–1.10)	0.790	64.0	0.062	0.341
	No	1.03 (0.88–1.21)	0.712	79.8	< 0.001	

*BMI body mass index, *CI* confidence interval, *CVD* cardiovascular disease, *DM* diabetes mellitus, *FFQ* food frequency questionnaire, *PA* physical activity, *RR* relative risk

### Stroke and stroke mortality

**Fig. 3:**
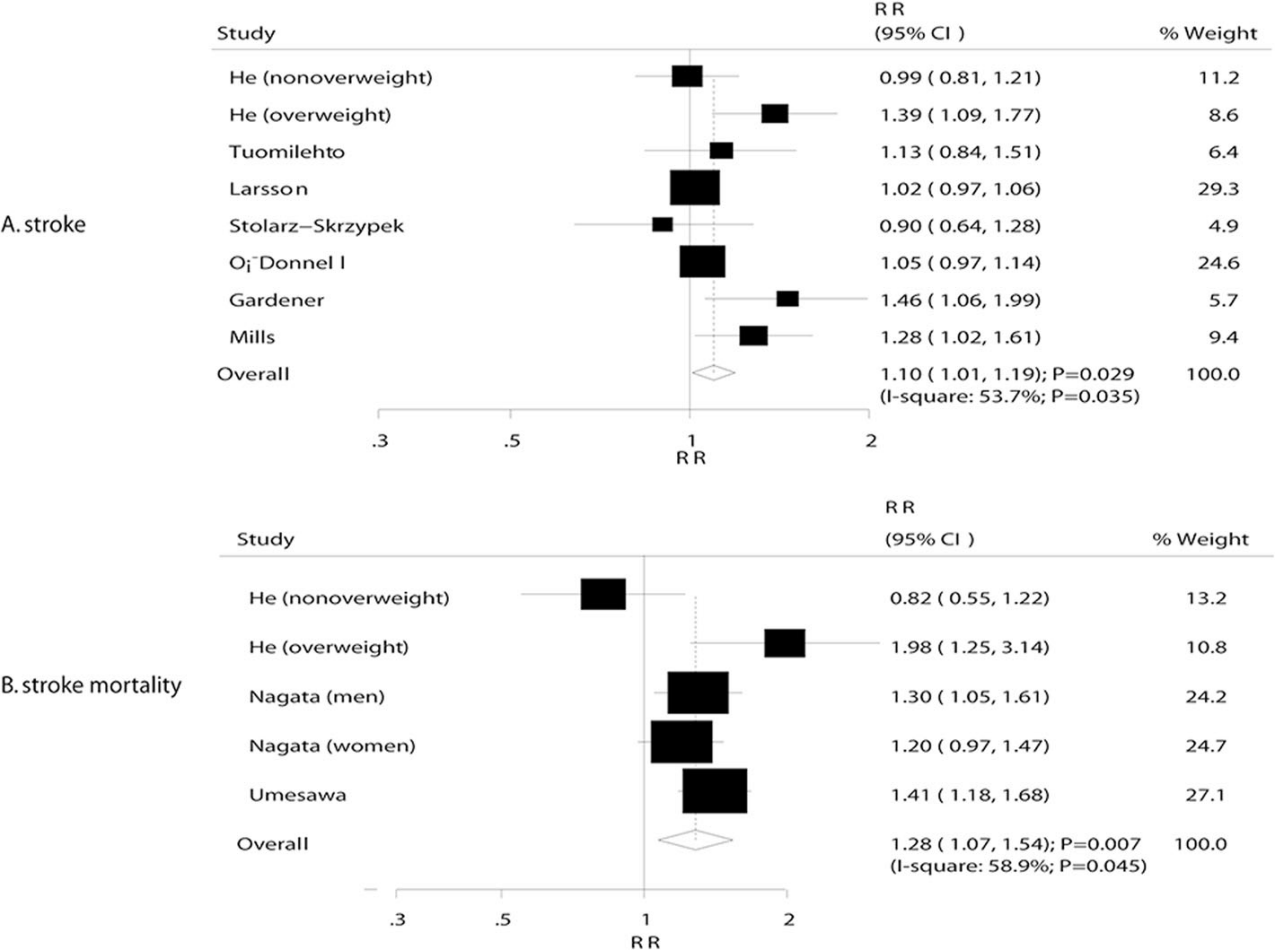
**a** Association between sodium intake and stroke. **b** Association between sodium intake and stroke mortality

A total of 7 studies reported an association between sodium intake and stroke, and 3 studies reported the association of sodium intake and stroke mortality. Pooled analysis of stroke and stroke mortality indicated that a 100 mmol increment per day in sodium intake exerted a harmful effect (stroke: RR, 1.10; 95% CI, 1.01–1.19; *P* = 0.029, Fig. 3a; stroke mortality: RR, 1.28; 95% CI, 1.07–1.54; *P* = 0.007, Fig. 3b). Heterogeneity was observed in the magnitude of the effect across the studies (I^2^ = 53.7%, *P* = 0.035 for stroke; I^2^ = 58.9%, *P* = 0.045 for stroke mortality). However, the conclusion was not affected by excluding any specific study after sequential exclusion of each study from all the pooled analyses. Furthermore, low (RR: 1.25; 95% CI: 0.85–1.85; *P* = 0.260), moderate (RR: 1.11; 95% CI: 1.00–1.24; *P* = 0.058), and heavy (RR: 1.02; 95% CI: 0.93–1.11; *P* = 0.720) sodium intake did demonstrate any effect on the subsequent stroke risk (Table 2). In addition, low sodium intake did not affect the stroke mortality (RR: 1.20; 95% CI: 0.96–1.50; *P* = 0.117), while moderate (RR: 1.50; 95% CI: 1.20–1.88; *P* < 0.001) and heavy (RR: 1.81; 95% CI: 1.29–2.55; *P* = 0.001) sodium intake was associated with a high risk of stroke mortality (Table 2). In addition, subgroup analysis suggested that a 100 mmol per day increment in sodium intake was associated with an increased risk of stroke if the sample size was < 10,000 (RR: 1.18; 95% CI: 1.02–1.36; *P* = 0.029), the proportion of males was < 60.0% (RR: 1.18; 95% CI: 1.02–1.36; *P* = 0.029), the study adjusted for BMI (RR: 1.10; 95% CI: 1.01–1.19; *P* = 0.029), smoking status (RR: 1.13; 95% CI: 1.00–1.28; *P* = 0.048), and PA(RR: 1.11; 95% CI: 1.01–1.22; *P* = 0.026), and the study not adjusted for the level of potassium (RR: 1.16; 95% CI: 1.02–1.33; P = 0.029) (Table 5). The subgroup analysis for stroke mortality was not conducted due to the small number of studies included in this investigation on the association of sodium intake and stroke mortality.

**Table 5:** Subgroup analyses for stroke

Factor	Subgroup	RR and 95% CI	*P* value	Heterogeneity (%)	*P* value for heterogeneity	*P* value between subgroups
Publication year	Before 2010	1.09 (0.95–1.24)	0.211	54.5	0.086	0.248
	2010 or after	1.14 (0.96–1.35)	0.127	58.2	0.067	
Sample size	≥ 10,000	1.03 (0.99–1.07)	0.181	0.0	0.537	0.020
	< 10,000	1.18 (1.02–1.36)	0.029	46.5	0.096	
Percentage male (%)	≥ 60.0	1.03 (0.99–1.07)	0.181	0.0	0.537	0.020
	< 60.0	1.18 (1.02–1.36)	0.029	46.5	0.096	
Assessment of exposure	FFQ	1.19 (0.95–1.50)	0.125	76.0	0.016	0.503
	24 h urine collection	1.08 (0.96–1.21)	0.190	36.8	0.176	
Follow-up duration (years)	≥ 10.0	1.15 (0.97–1.37)	0.116	72.4	0.012	0.453
	< 10.0	1.08 (0.98–1.20)	0.136	18.2	0.300	
Adjusted BMI	Yes	1.10 (1.01–1.19)	0.029	53.7	0.035	–
	No	–	–	–	–	
Adjusted smoking	Yes	1.13 (1.00–1.28)	0.048	60.2	0.020	0.865
	No	1.05 (0.97–1.14)	0.236	–	–	
Adjusted alcohol	Yes	1.14 (0.99–1.31)	0.072	66.2	0.011	0.744
	No	1.06 (0.98–1.14)	0.174	0.0	0.636	
Adjusted Previous CVD	Yes	1.09 (0.99–1.20)	0.074	64.4	0.038	0.395
	No	1.10 (0.91–1.32)	0.317	49.7	0.114	
Adjusted DM	Yes	1.14 (0.99–1.31)	0.072	66.2	0.011	0.744
	No	1.06 (0.98–1.14)	0.174	0.0	0.636	
Adjusted PA	Yes	1.11 (1.01–1.22)	0.026	64.6	0.015	0.892
	No	1.03 (0.82–1.29)	0.811	0.0	0.326	
Adjusted potassium	Yes	1.04 (0.96–1.13)	0.309	0.0	0.396	0.963
	No	1.16 (1.02–1.33)	0.029	65.2	0.013	

*BMI body mass index, *CI* confidence interval, *CVD* cardiovascular disease, *DM* diabetes mellitus, *FFQ* food frequency questionnaire, *PA* physical activity, *RR* relative risk

### Publication bias

**Fig. 4:**
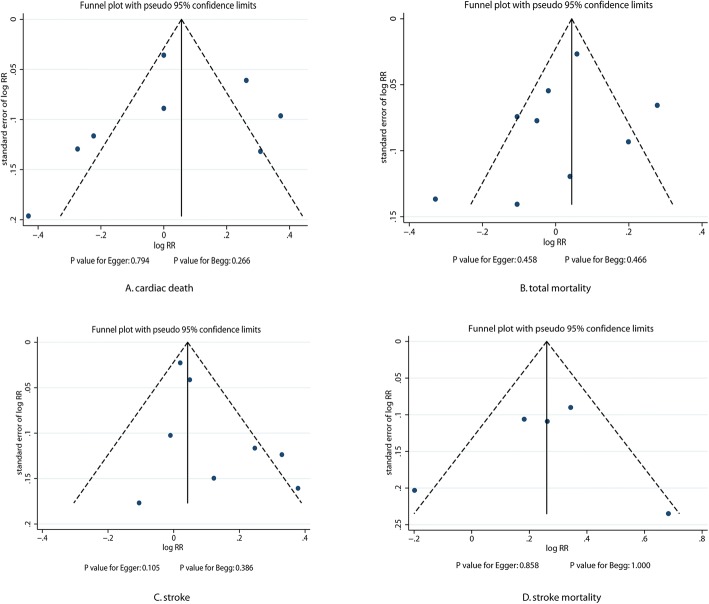
Publication bias tests for cardiac death, total mortality, stroke, and stroke mortality. Each point represents an independent study of the indicated association

The review of the funnel plots did not exclude the potential for publication bias for cardiac death, total mortality, stroke, and stroke mortality (Fig. 4). The Egger and Begg test’s results did not show any evidence of publication bias for cardiac death (*P*-value for Egger: 0.794; *P*-value for Begg: 0.266), total mortality (*P*-value for Egger: 458; *P*-value for Begg: 0.466), stroke (P-value for Egger: 0.105; *P*-value for Begg: 0.386), and stroke mortality (*P*-value for Egger: 0.858; *P*-value for Begg: 1.000).

## Discussion

The current study included the prospective cohort studies and explored the possible correlations between sodium intake and the outcomes of cardiac death, total mortality, stroke, and stroke mortality. This quantitative meta-analysis included a total of 205,575 individuals from 16 prospective cohort studies with a broad range of populations. The meta-analysis findings suggested that an increment of 100 mmol/day in sodium intake did not affect the incidence of cardiac death and total mortality. However, a 100 mmol per day increment in sodium intake significantly increased the risk of stroke and stroke mortality. Furthermore, parameters such as sample size, the proportion of males, assessment of exposure, follow-up duration, and several other adjusted factors were found to be associated with the correlation between sodium intake and major cardiovascular outcomes.

A previous meta-analysis suggested that high sodium intake was associated with a significantly increased risk of stroke and total cardiovascular diseases [44]. However, other 2 meta-analysis studies based on randomized controlled trials suggested that the reduced dietary salt did not affect the cardiovascular morbidity or mortality [45, 46]. The inherent limitation of this study included shorter duration of follow-up period than that required to show a clinical benefit, especially when the rate of events was lower than expected, which without any statistically significant difference. Furthermore, reduced dietary sodium intake seems to be associated with the degree of control achieved. Finally, the range of sodium intake and the cut-off values for the three categories differed among various studies. Therefore, we conducted a dose-response meta-analysis of these prospective studies for evaluating the optimal dose of sodium intake.

The current findings were in agreement with a recently published large cohort study conducted in Manhattan [40]. Our meta-analysis study included 2657 individuals and found that the participants who consumed > 4000 mg/d sodium demonstrated a 159% increased risk of stroke. Also, the risk percentage of stroke was increased by 17% for each increase in 500 mg/d. He et al. suggested that high sodium intake was strongly and independently associated with an increased risk of stroke mortality in overweight individuals, thereby significantly increasing the risk of total mortality [33]. Also, the current study indicated that increased sodium intake significantly elevated the risk of stroke and stroke mortality, while no effect on cardiac death and total mortality was demonstrated, which might be attributed to the increased blood pressure and hypertension due to high sodium levels by stiffening the endothelial cells, thickening and narrowing of resistance arteries, and blocking of nitric oxide synthesis [47].

The current study did not demonstrate a significant difference between 100 mmol increments of sodium intake per day and the risk of cardiac death. However, inconsistent results were reported by individual studies. O’Donnell et al. indicated that sodium excretion > 7 g/d was associated with an increased risk of cardiac death and coronary heart disease (CHD) as compared to sodium excretion of 4–5.99 g/d [39]. Furthermore, Tunstall­Pedoe et al. suggested that high sodium intake significantly increased the cardiac death and CHD by 36% and 34%, respectively [32]. This phenomenon might be attributed to the inclusion of other prospective studies encompassing general individuals; however, these 2 studies specifically included individuals with high risk of cardiovascular disease, rendering them susceptible to extreme sodium intake.

Subgroup analysis suggested that a 100 mmol increment of sodium intake per day was associated with cardiac death reduction if the study was not adjusted for BMI; also, the risk of cardiac death was increased significantly if the follow-up duration was ≥10 years. In addition, the risk of total mortality was increased if the sample size was ≥10,000 and percentage male was ≥60.0%. Finally, and increased sodium intake by 100 mmol per day was associated with an elevated risk of stroke if the sample size was < 10,000, the percentage of males was < 60.0%, the study adjusted for BMI, smoking status, PA, and the study not adjusted for potassium level. However, these conclusions might be unreliable due to the inclusion of small cohorts in each subset. Therefore, this study provided a relative result as well as a synthetic and comprehensive review.

The three strengths of our study should be highlighted. Firstly, only prospective cohort studies were included, which eliminated the selection as well as recall bias and could be a concern for retrospective case-control studies. Secondly, a large sample size allowed us to quantitatively assess the association of sodium intake with the risk of cardiovascular morbidity and mortality, which in turn, demonstrated that our findings are potentially more robust than any individual studies. Thirdly, the pooled analysis included a wide range of sodium intake levels, which subsequently allowed an accurate assessment of the relation of sodium intake and major cardiovascular risk outcomes.

Nevertheless, the present study had some limitations as follows: (1) the adjusted models used in the included studies are different, and these factors might play a critical role in the development of CVDs; (2) the minimal intake of sodium in individual study varied, which might introduce uncontrolled biases and potential heterogeneity; (3) heterogeneity across included studies was high, and hence, the results of publication bias test were not reliable; (4) high heterogeneity was not investigated by subgroup analysis due to the minimal intake of sodium and cutoff value, and the adjusted factors were not consistent among included studies; (5) the meta-analysis used pooled data due to the unavailability of individual data, which restricted a detailed and relevant analysis in order to obtain comprehensive results.

## Conclusions

In conclusion, the results of this study suggested that increased sodium intake might play a major role in the risk of stroke morbidity and mortality. However, the increased sodium intake did not have a significant effect on cardiac death and total mortality. Nevertheless, future studies focusing on specific populations for analyzing the secondary prevention of major cardiovascular outcomes are warranted.

## Abbreviations

BMI: Body mass index
CHD: Coronary heart disease
CIs: Confidence intervals
CVD: Cardiovascular diseases
HR: Hazard ratio
NOS: Newcastle–Ottawa Scale
OR: Odds ratio
RR: Relative risk
